# Disease Control through Voluntary Vaccination Decisions Based on the Smoothed Best Response

**DOI:** 10.1155/2014/825734

**Published:** 2014-02-16

**Authors:** Fei Xu, Ross Cressman

**Affiliations:** Department of Mathematics, Wilfrid Laurier University, Waterloo, ON, Canada N2L 3C5

## Abstract

We investigate game-theory based decisions on vaccination uptake and its effects on the spread of an epidemic with nonlinear incidence rate. It is assumed that each individual's decision approximates his/her best response (called smoothed best response) in that this person chooses to take the vaccine based on its cost-benefit analysis. The basic reproduction number of the resultant epidemic model is calculated and used to characterize the existence and stability of the disease-free and endemic equilibria of the model. The effects on the spread and control of the epidemic are revealed in terms of the sensitivity of the response to changes in costs and benefits, in the “cost” of the vaccination, and in the proportion of susceptible individuals who are faced with the decision of whether or not to be vaccinated per unit time. The effects of the best response decision rule are also analyzed and compared to those of the smoothed best response. Our study shows that, when there is a perceived cost to take the vaccine, the smoothed best response is more effective in controlling the epidemic. However, when this cost is 0, the best response is the more efficient control.

## 1. Introduction

In modern society, infectious diseases threaten millions of people's lives each year and, as such, controlling the spread of these diseases is essential. As one of the effective control strategies, vaccination against infectious diseases has been widely used to slow down or eliminate their spread [1–4]. Recent investigations of theoretical models based on different vaccination policies [2, 3] indicate that there are many ways an effective vaccine can be used to control an epidemic.

These theoretical models often consider the “cost” to get vaccinated. Besides the actual monetary cost of the vaccine, there are potential risks to being vaccinated. Thus people making rational decisions may avoid vaccinations when the perceived cost of taking the vaccine is higher than its benefits. That is, individual decisions about the vaccination uptake might follow a cost-benefit analysis. Thus, the analysis of the effect of voluntary vaccination decisions is becoming increasingly important as people are now able to obtain up-to-date information about the spread of an epidemic as well as about the cost of vaccination.

The aim of this paper is to model how individuals implement their rational decisions on vaccine uptake and investigate the effects of these decisions on the spread and control of the epidemic. On one hand, susceptibles have the risk of being infected. On the other hand, due to the perceived risk of vaccine side effects, susceptible individuals might choose not to receive the vaccination. During an epidemic, a susceptible individual has to make a choice based on the risk of being vaccinated and the risk of getting infected. We use game theory to model this situation since this theory studies how individuals optimize their behavior given their net benefits and the behavior of others (i.e., how individuals make rational decisions). Since the probability that a susceptible individual gets infected decreases as the vaccination level of the population increases, rational decisions may lead to a reduced number of vaccination intakes whereby rational individuals rely on others to maintain the vaccination level of the population. This situation is also known as “free riding” [5]. However, this free riding strategy is not optimal to control the disease spread in the long run. That is, these rational decisions will lead to an increase in the number of susceptibles, followed by an eventual increase in the number of infected. In this work, we are particularly interested in the “degree of rationality” of the susceptible individuals and the corresponding effects on the long-term infection rates as well as the control and spread of the disease.

**Figure 1:**
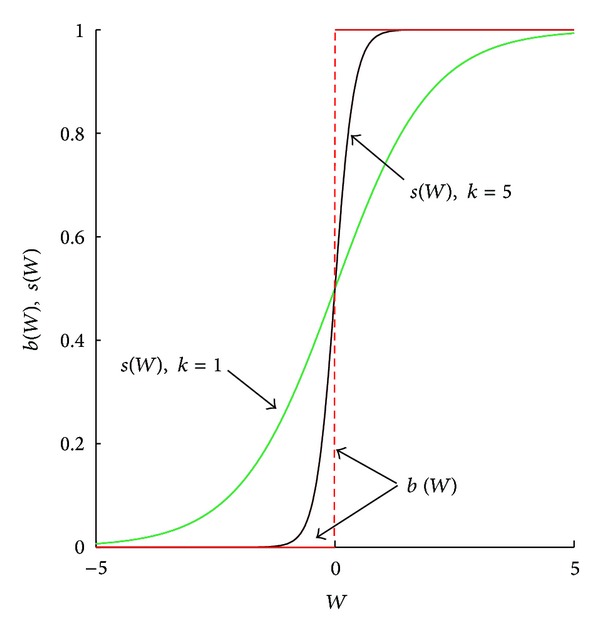
Graphs of best response function *b*(*W*) and smoothed best response function *s*(*W*) for degree of rationality (or sensitivity) *k* = 1 and *k* = 5.

To model such decisions, we use methods from evolutionary game theory whereby strategies that have higher net benefits increase in the population. One such method, called the best response [6], assumes that all individuals who are faced with a decision choose the strategy with the highest payoff. In our model, this means a susceptible will choose to be vaccinated if the risk of infection outweighs the cost of vaccination. The best response requires the decision maker to have a precise knowledge of these costs and benefits. Instead, we concentrate on a second method, called the smoothed best response [7], as the basis for individual decisions. Here, individuals with lower payoff switch to the best strategy with a certain probability. If payoff differences are large, they are almost certain to switch but this probability decreases as the payoffs become closer to each other. This may reflect that information on net payoffs are not precise. Alternatively, in our interpretation, how quickly switching probabilities change (as a function of payoff differences) measures the degree of rationality for the model (cf.  Figure 1).

In this paper, we construct and analyze an evolutionary game-theoretic epidemic model to study the effects of a game-theory based vaccination decision on the spread and control of an epidemic. As we will see, evolutionary dynamics based on the smoothed best response are more effective at controlling the disease than those based on the best response.

Similar methods based on other evolutionary dynamics (such as the replicator equation or imitative dynamics) are commonly used to show that observed behavior of animal species can be predicted by assuming individuals act so as to maximize their per capita growth rates in ecology systems (e.g., [8, 9]). Although such dynamics can also be interpreted as resulting from rational decision making, these decisions are typically assumed to come from observing the behavior of a randomly chosen individual in the population and then deciding whether to imitate this behavior. This contrasts with our model whereby decisions are made through knowledge of the overall costs and benefits of the system. In the extensive literature on the effects of individual rational behavior on the spread of an epidemic summarized in the following paragraph, either the models do not take an evolutionary game theory approach or the evolutionary dynamics is based on imitative behavior. Our model then extends the evolutionary approach to what we feel are more realistic assumptions on how individuals implement their rational decisions.

The effects of individual rational behavior on epidemic models that include (voluntary) vaccination have been investigated in the literature. For example, Fine and Clarkson studied the rational decisions of well-informed individuals on the vaccine uptake and their corresponding effects on infection control [10]. By developing a game-theory based epidemic model, Bauch and Earn investigated the consequences of voluntary vaccination strategies for childhood diseases with the assumption that self-interested parents may choose to avoid vaccination due to possible side effects [11]. Bauch investigated individual vaccinating decisions with the assumption that the susceptibles behave strategically in accordance with imitation dynamics and studied the dependence of epidemic prevalence and coverage of vaccination on these strategic decisions [12]. Reluga et al. studied population-level demand for vaccines and the decisions of individuals to avoid infection by constructing and analyzing a game-theoretical model [13]. Perisic and Bauch studied the influences of individual behavior on the epidemic transmission in contact networks and obtained three possible outcomes associated with the long run number of vaccinated individuals and epidemic size [14]. By designing and analyzing a game-theoretic model, Perisic and Bauch investigated the behavior-infection dynamics on social contact networks [15]. Combining Markov decision process theory and game theory, Reluga and Galvani investigated the payoffs of individuals and communities in vaccination games and studied their effects on epidemic control [16]. Using a model based on evolutionary game theory, Schimit and Monteiro considered the interplay between public health actions and personal decisions during an epidemic [17]. Mbah et al. considered the epidemic spread through an epidemiological network and the effects of imitation behavior of individuals on the vaccination uptake using evolutionary game theory [18]. Zhang et al. constructed and analyzed two simple models to investigate the “double-edged sword” effect that rational decision making has on public health condition [19]. Using an evolutionary game-theory based strategy, Poletti et al. studied several patterns of risk perception and information diffusion during an epidemic spread [20]. Chen constructed a mathematical model to investigate the strategic behaviors of individuals to avoid public places during an epidemic [21]. Shim et al. investigated how the avoidance of Measles-Mumps-Rubella vaccination due to the perceived side effects is related to the spread of this disease [22].

The paper is organized as follows. In Section 2, we present the epidemic model with the (smoothed) best response vaccination dynamics included. The existence and stability of the disease free and endemic equilibria of the model are analyzed in Section 3.  Section 4 is devoted to discuss the results and their significance with detailed numerical simulations. Finally, conclusions are given in Section 5.

## 2. The Epidemic Model with Voluntary Vaccination

We assume that the total population size at time *t* is classified into four groups with respect to their epidemiological status. These groups are susceptibles (*S*(*t*)), infected (*I*(*t*)), recovered (*R*(*t*)), and vaccinated (*V*(*t*)). New susceptible individuals enter the subgroup *S*(*t*) at a constant rate of *A* through birth or immigration. The death rate *d* is assumed to be constant for all four groups. Individuals leave subgroup *S*(*t*) through death, infection, and vaccination. We assume that susceptible individuals contract the disease with incidence rate *βI*/(*S*+*I*+*R*+*V*)^*q*^, where *q* ∈ [0, 1] is a fixed parameter. This includes the two most common incidence rates used in the literature, namely, the standard incidence rate (*q* = 1) and the bilinear incidence rate (*q* = 0) [23].

If an individual's decision on taking the vaccination follows a cost-benefit analysis, the vaccination rate will be a function *φ*(*S*, *I*, *R*, *V*) of the sizes of these four groups. Susceptible individuals who acquire infection enter the infective group, and infective individuals exit this group by death (with rate *d*) or recovery (with rate *r*). Recovered infective individuals enter the recovered group and susceptible individuals who get vaccines enter the vaccinated group. We assume that both naturally acquired immunity (through infection) and artificially acquired immunity (through vaccination) are permanent; that is, individuals in the recovered group or in the vaccinated group do not leave their groups to enter other groups.

The epidemic model with game-theory based vaccination decisions is then given by the following system of differential equations:

(1) $$\begin{array}{c} \dot{S}=A-\frac{\beta SI}{{\left(S+I+R+V\right)}^{q}}-dS-S\varphi \left(S,I,R,V\right),\\ \dot{I}=\frac{\beta SI}{{\left(S+I+R+V\right)}^{q}}-rI-dI,\\ \dot{R}=rI-dR,\\ \dot{V}=S\varphi \left(S,I,R,V\right)-dV. \end{array}$$

The variables in system (1) describe the population sizes of each epidemiological group, and thus we assume that they are all nonnegative. In the following, we will investigate the dynamical behavior of system (1) in the biologically feasible region Γ given by

(2) $$\begin{array}{c} \Gamma =\left\{\left(S,I,R,V\right)\in {\mathbb{R}}_{+}^{4}:S+I+R+V\leq \frac{A}{d}\right\}. \end{array}$$

Notice that the total population size *N* = *S* + *I* + *R* + *V* satisfies $\dot{N}=A-dN$, indicating that *N*(*t*) = *A*/*d* + e^−*dt*^(*N*(0) − *A*/*d*). Hence, the region Γ is positively invariant and globally attracting. In this work, we only investigate the dynamic behavior of the model with initial conditions (*S*(0), *I*(0), *R*(0), *V*(0)) ∈ Γ.

### 2.1. Game-Theoretic Vaccination Decisions

Vaccination is an effective approach to prevent disease infection. However, there is a cost to being vaccinated, including the risk of infection by taking the vaccine and perhaps some financial cost as well. If each individual is able to make their own decision on whether or not to be vaccinated, then this behavior can be modeled using game theory. If (unvaccinated) susceptible individuals contract the disease with incidence rate *βI*/(*S*+*I*+*R*+*V*)^*q*^, for *q* ∈ [0, 1], then this value can be used as the payoff benefit obtained by an individual who takes the vaccine. Here it is assumed that the vaccination is effective (i.e., vaccinated individuals are not susceptible). For simplicity, we also assume that the perceived cost of taking the vaccination is a constant *α* for each individual. Then an individual also incurs a payoff loss of −*α* from taking the vaccination. The total payoff of an individual who is vaccinated compared to one who is not is then given by *W* = *βI*/(*S*+*I*+*R*+*V*)^*q*^ − *α*.

Recently, the logistic equation [24] and its inverse (the logit map [25]) have been used in evolutionary game theory to describe a particular type of rational decision making called the smoothed best response correspondence [7]. The logistic equation [24] takes the form of a sigmoid function, which can be written as

(3) $$\begin{array}{c} ℒ\left(x\right)=\frac{{\text{e}}^{x}}{{\text{e}}^{x}+{\text{e}}^{a}}. \end{array}$$

Logistic equations are widely used in statistics and have broad applications in chemistry, physics, biology, and economics. For game-theoretic applications with two strategies, the smoothed best response function has the form

(4) $$\begin{array}{c} s\left(W_{p},W_{n}\right)=\frac{{\text{e}}^{kW_{p}}}{{\text{e}}^{kW_{p}}+{\text{e}}^{kW_{n}}}, \end{array}$$

where *W*_*p*_ = *βI*/(*S*+*I*+*R*+*V*)^*q*^ and *W*_*n*_ = *α* denote the positive and negative payoffs, respectively, and *k* ≥ 0 is constant. In our context, *s* is interpreted as the probability a susceptible individual decides to take the vaccine when faced with this decision. Notice that *ℒ*(*x*) and *s* are both in the interval (0, 1).

The smoothed best response function *s*(*W*_*p*_, *W*_*n*_) of the vaccination game can also be expressed as the function of the total payoff of the game *W* = *W*_*p*_ − *W*_*n*_; that is,

(5) $$\begin{array}{c} s=\frac{{\text{e}}^{k\beta I/{\left(S+I+R+V\right)}^{q}}}{{\text{e}}^{k\beta I/{\left(S+I+R+V\right)}^{q}}+{\text{e}}^{k\alpha }}=\frac{\tanh\left(\left(1/2\right)kW\right)+1}{2}. \end{array}$$

When *k* = 0, the individual is indifferent and decides to be vaccinated half the time irrespective of costs and benefits. For positive *k*, almost all individuals will choose to be vaccinated when benefits greatly exceed costs (i.e., for large *W*) but very few will be vaccinated when costs are much higher than benefits. With an increase in *k*, the sensitivity of the response to the changes in differences in costs and benefits when *W* is close to zero becomes more pronounced (Figure 1). That is, the “right” choice is more likely to be made with respect to the cost-benefit analysis as *k* increases. For the extreme situation when *k* → *∞*, the smoothed best response approaches the best response; that is,

(6) $$\begin{array}{c} b=\{\begin{array}{cc} 0 & \text{if}\;W<0\\ \left[0,1\right] & \text{if}\;W=0\\ 1 & \text{if}\;W>0. \end{array} \end{array}$$

Both the classic best response [6] and the smoothed best response [7] have been widely used to address rational decision making of an individual [9, 18, 19, 26]. The smoothed and nonsmoothed best response behave differently when *W* → 0. For the classical best response function, the value of *b* is either 1 or 0, determined by the sign of *W* even if *W* is close to 0. In this case, individual decisions are extremely sensitive to the payoff difference. When benefits and costs are equal (i.e., the total payoff is *W* = 0), *b* can be any value in the interval [0, 1]. For the smoothed best response function, *s*(*W*) is a continuous function on (−*∞*, *∞*), increasing from 0 to 1. In particular, lim⁡_*W*→0_ *s*(*W*) = *s*(0) = 1/2, implying that the probability of picking either strategy is approximately 1/2 when *W* is small (and, when *W* = 0, each strategy is equally likely to be chosen). The relation between smoothed and nonsmoothed best response is shown in Figure 1. Notice that parameter *k* is proportional to the slope of the curve *s*(*W*) at *W* = 0.

Under the smoothed best response, the per individual rate of vaccination uptake is then given as *φ*(*S*, *I*, *R*, *V*) = *ϕs*(*W*) = *ϕ*(e^*kβI*/(*S*+*I*+*R*+*V*)^*q*^^/(e^*kβI*/(*S*+*I*+*R*+*V*)^*q*^^ + e^*kα*^)). Here *ϕ* is a constant between 0 and 1 indicating the proportion of susceptible individuals who are faced with the decision of whether or not to be vaccinated per unit time. Since max⁡(*φ*(*S*, *I*, *R*, *V*)) = lim⁡_*W*→*∞*_ *ϕs*(*W*) = *ϕ*, *ϕ* is also the proportion of the susceptibles who take the vaccine per unit time when the total payoff of the vaccination is quite high.

## 3. The Disease Free and Endemic Equilibria: Existence and Stability

System (1) always admits a disease free equilibrium *E*_0_ = (*S*_0_*, 0, 0, *V*_0_*), where

(7) $$\begin{array}{c} S_{0}^{*}=\frac{A}{d+\phi s}=\frac{2A}{2d+\phi \left(1-\tanh\left(\left(1/2\right)k\alpha \right)\right)},\\ V_{0}^{*}=\frac{\phi A\left(1-\tanh\left(\left(1/2\right)k\alpha \right)\right)}{d\left(2d+\phi \left(1-\tanh\left(\left(1/2\right)k\alpha \right)\right)\right)}, \end{array}$$

since *s* = (tanh(−(1/2)*kα*) + 1)/2 = (1 − tanh((1/2)*kα*))/2 when *W*_*p*_ = 0. Note that *S*_0_* and *V*_0_* are both positive since 0 < tanh((1/2)*kα*) < 1 for positive *k* and *α*. The basic reproduction number *ℛ*_0_ (i.e., the expected number of infected individuals generated over its lifetime by the introduction of a single infected at the disease free equilibrium) plays an important role in the stability of *E*_0_. For system (1), *ℛ*_0_ can be obtained by using the next generation method [27] and is given by (see the Appendix)

(8) $$\begin{array}{c} {ℛ}_{0}=\frac{2\beta A}{{\left(A/d\right)}^{q}\left(r+d\right)\left(2d+\phi \left(1-\tanh\left(\left(1/2\right)k\alpha \right)\right)\right)}. \end{array}$$

**Theorem 1**

If *ℛ*_0_ < 1, the disease free equilibrium *E*_0_ of model (1) is the only equilibrium and it is locally asymptotically stable. If *ℛ*_0_ > 1, *E*_0_ is unstable.

**Proof**

The local stability of the disease-free equilibrium *E*_0_ is determined by the Jacobian matrix *J*_0_ of system (1) at *E*_0_, which is given by (9)

(9) [L2−(−4−kϕ+kϕ  (tanh((1/2)kα))2)βA2(A/d)qL000−2βA(A/d)qL−d−r000r−d0−12ϕ(tanh(12kα)−1)(1/2)ϕA(−1+(tanh((1/2)kα))2)kβ  (A/d)qL0−d],

where *L* = *ϕ*  (tanh((1/2)*kα*) − 1) − 2*d* < 0. We notice that −*d* is an eigenvalue of *J*_0_ with multiplicity 2, and the remaining two eigenvalues are also eigenvalues of the 2 × 2 matrix

(10) $$\begin{array}{c} \left[\begin{array}{cc} \frac{L}{2} & -\frac{\left(-4-k\phi +k\phi \;{\left(\tanh\left(\left(1/2\right)k\alpha \right)\right)}^{2}\right)\beta A}{2{\left(A/d\right)}^{q}L}\\ 0 & -\frac{2\beta A}{{\left(A/d\right)}^{q}L}-d-r \end{array}\right]. \end{array}$$

Since *L* < 0, all eigenvalues of *J*_0_ are negative if and only if −2*βA*/((*A*/*d*)^*q*^ *L*) − *d* − *r* < 0, which is equivalent to *ℛ*_0_ < 1. Thus, the disease free equilibrium *E*_0_ is locally asymptotically stable when *ℛ*_0_ < 1. Furthermore, −2*βA*/((*A*/*d*)^*q*^ *L*) − *d* − *r* > 0 when *ℛ*_0_ > 1, indicating that the disease free equilibrium *E*_0_ is unstable in this case.

Any other equilibrium *E*_1_  (*S*_1_*, *I*_1_*, *R*_1_*, *V*_1_*) of system (1) has *I*_1_* ≠ 0. From this, it follows that *E*_1_ has the form

(11) $$\begin{array}{c} S_{1}^{*}=\frac{{\left(A/d\right)}^{q}\left(r+d\right)}{\beta },\\ I_{1}^{*}=\frac{{\left(A/d\right)}^{q}\left(k\alpha +2Q\right)}{k\beta },\\ R_{1}^{*}=\frac{{\left(A/d\right)}^{q}r\left(k\alpha +2Q\right)}{k\beta d},\\ V_{1}^{*}=\frac{Ak\beta -{\left(A/d\right)}^{q}\left(r+d\right)\left(dk+2Q+k\alpha \right)}{k\beta d}, \end{array}$$

where *Q* is the root of the following function:

(12) $$\begin{array}{c} P_{1}{\text{e}}^{2x}x+P_{2}{\text{e}}^{2x}+P_{3}x+P_{4}=0. \end{array}$$

Here,

(13) P1=2(Ad)q(r+d),P2=(Ad)qk(r+d)(d+α)−Akβ+kϕ(Ad)q(r+d),P3=2(Ad)q(r+d)=P1,P4=(Ad)qk(r+d)(d+α)−Akβ=P2−kϕ(Ad)q(r+d).

From (12) and (13), *Q* satisfies *P*_2_/((*A*/*d*)^*q*^ *kϕ*(*r* + *d*)) + (2/(*ϕk*))*Q* = 1/(e^2*Q*^ + 1) and so it is the intersection of the following two functions:

(14) $$\begin{array}{c} f_{1}\left(x\right)=\frac{1}{{\text{e}}^{2x}+1},\\ f_{2}\left(x\right)=\frac{P_{2}}{{\left(A/d\right)}^{q}k\phi \left(r+d\right)}+\frac{2}{\phi k}x. \end{array}$$

Here *f*_1_(*x*) is a decreasing function and *f*_2_(*x*) is a linear function with slope 2/(*ϕk*). By substitution, *f*_1_(−*kα*/2) = *f*_2_(−*kα*/2) when *ℛ*_0_ = 1 (i.e., *E*_1_ = *E*_0_ since *Q* = −*kα*/2 and so *I*_1_* = 0 = *R*_1_*).

When *ℛ*_0_ < 1, we have *f*_1_(−*kα*/2) < *f*_2_(−*kα*/2). Thus the point of intersection of the curve *y* = *f*_1_(*x*) and the line *y* = *f*_2_(*x*) is to the left of *x* = −*kα*/2 (i.e., *Q* < (−*kα*/2)). Thus, *I*_1_* < 0. In summary, if *ℛ*_0_ < 1, there is no biologically feasible solution to (12) and (13) for which *E*_1_ has all nonnegative components.

**Theorem 2**

When *ℛ*_0_ > 1, the endemic equilibrium *E*_1_  (*S*_1_*, *I*_1_*, *R*_1_*, *V*_1_*) exists in Γ and it is locally asymptotically stable.

**Proof**

Suppose that *ℛ*_0_ > 1.

*Existence.* From the proof of Theorem 1, *f*_1_(−*kα*/2) > *f*_2_(−*kα*/2). Furthermore, −*kα*/2 < *k*(*Aβ* − (*A*/*d*)^*q*^(*r* + *d*)(*d* + *α*))/(2(*A*/*d*)^*q*^(*r* + *d*)) and *f*_1_(*k*(*Aβ* − (*A*/*d*)^*q*^(*r* + *d*)(*d* + *α*))/(2(*A*/*d*)^*q*^(*r* + *d*))) < *f*_2_(*k*(*Aβ* − (*A*/*d*)^*q*^(*r* + *d*)(*d* + *α*))/(2(*A*/*d*)^*q*^(*r* + *d*))) = 1. Since *f*_1_(*x*) is a decreasing function and *f*_2_(*x*) is an increasing function, the solution *Q* to (12) is in the interval (−*kα*/2, *k*(*Aβ* − (*A*/*d*)^*q*^(*r* + *d*)(*d* + *α*))/(2(*A*/*d*)^*q*^(*r* + *d*))), indicating that *I*_1_* > 0, *R*_1_* > 0, *V*_1_* > 0. Notice that *S*_1_* = (*A*/*d*)^*q*^(*r* + *d*)/*β* > 0 and *S*_1_* + *I*_1_* + *R*_1_* + *V*_1_* = *A*/*d*. Hence, *E*_1_ is an endemic equilibrium of system (1) when *ℛ*_0_ > 1.

*Stability.* To prove the local stability of the endemic equilibrium, the Jacobian matrix *J*_1_ of system (1) at *E*_1_ is given by (15) (this linearization and the subsequent evaluation of the eigenvalues of *J*_1_ were obtained using MAPLE)

(15) $$\begin{array}{c} \left[\begin{array}{cccc} \frac{H\left(4{\left(\cosh\left(Q\right)\right)}^{2}+\phi k\right)}{4k\beta {\left(\cosh\left(Q\right)\right)}^{2}A}-d & \frac{H\left(4{\left(\cosh\left(Q\right)\right)}^{2}+\phi \;k\right)}{4k\beta {\left(\cosh\left(Q\right)\right)}^{2}A} & \frac{H\left(4{\left(\cosh\left(Q\right)\right)}^{2}+\phi k\right)}{4k\beta {\left(\cosh\left(Q\right)\right)}^{2}A} & \frac{H\left(4{\left(\cosh\left(Q\right)\right)}^{2}+\phi k\right)}{4k\beta {\left(\cosh\left(Q\right)\right)}^{2}A}\\ -\alpha -2\frac{Q}{k}-\frac{\phi \left(\tanh\left(Q\right)+1\right)}{2} & -\frac{\phi k\left(r+d\right)}{4{\left(\cosh\left(Q\right)\right)}^{2}}-r-d & & \\ \frac{2Q+\alpha k}{k}-\frac{H}{Ak\beta } & -\frac{H}{Ak\beta } & -\frac{H}{Ak\beta } & -\frac{H}{Ak\beta }\\ 0 & r & -d & 0\\ \frac{\phi \left(1+\tanh\left(Q\right)\right)}{2}-\frac{\phi H}{4\beta A{\left(\cosh\left(Q\right)\right)}^{2}} & \frac{\phi \left(r+d\right)k}{4{\left(\cosh\left(Q\right)\right)}^{2}}-\frac{\phi H}{4\beta A{\left(\cosh\left(Q\right)\right)}^{2}} & -\frac{\phi H}{4\beta A{\left(\cosh\left(Q\right)\right)}^{2}} & -d-\frac{\phi H}{4\beta A{\left(\cosh\left(Q\right)\right)}^{2}} \end{array}\right], \end{array}$$

where *H* = (*A*/*d*)^*q*^ *qd*(*r* + *d*)(2*Q* + *αk*). Two of the eigenvalues of *J*_1_ are *λ*_1_ = −*d* and *λ*_2_ = −*d*, and the other two eigenvalues, *λ*_3_ and *λ*_4_, are the roots of the following polynomial:

(16) λ2+(2Q+dk+αk)(e2Q+1)+ϕkk(e2Q+1)λ  +(d+r)(αk+2Q)(2e2Q+ϕke2Q+e4Q+1)k(e2Q+1)2.

Notice that when *ℛ*_0_ > 1, *Q* ∈ (−*kα*/2, *k*(*Aβ* − (*A*/*d*)^*q*^(*r* + *d*)(*d* + *α*))/(2(*A*/*d*)^*q*^(*r* + *d*))), which guarantees that ((2*Q* + *dk* + *αk*)(e^2*Q*^ + 1) + *ϕk*)/(*k*(e^2*Q*^ + 1)) > 0 and (*d* + *r*)(*αk* + 2*Q*)(2e^2*Q*^ + *ϕk*e^2*Q*^ + e^4*Q*^ + 1)/*k*(e^2*Q*^+1)^2^) > 0. Thus, the roots of polynomial (16) have negative real parts. Hence, the endemic equilibrium *E*_1_ of system (1) is locally asymptotically stable for *ℛ*_0_ > 1 (and does not exist as a biologically feasible equilibrium when *ℛ*_0_ < 1).

## 4. Discussions

From the theory developed in the preceding section, we see that the disease free equilibrium *E*_0_ is locally asymptotically stable if and only if *ℛ*_0_ < 1, where

(17) $$\begin{array}{c} {ℛ}_{0}=\frac{2\beta A}{{\left(A/d\right)}^{q}\left(r+d\right)\left(2d+\phi \left(1-\tanh\left(\left(1/2\right)k\alpha \right)\right)\right)}. \end{array}$$

**Figure 2:**
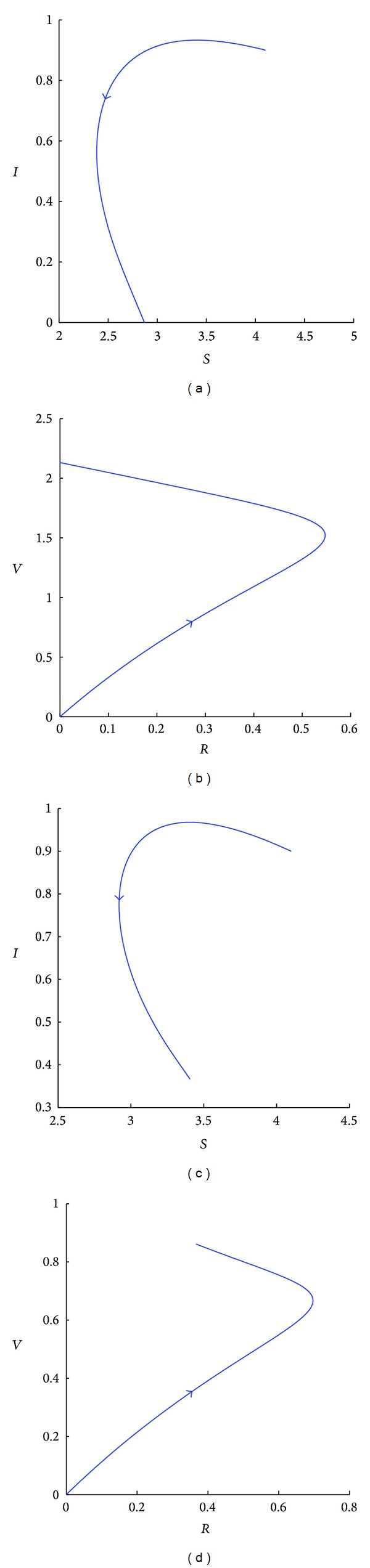
Simulated phase portraits of system (1) for *A* = 0.5, *β* = 0.25, *q* = 0.9, *d* = 0.1, *α* = 0.01, *r* = 0.1, and *k* = 2, projected on the *S* − *I* plane (a, c) and on the *R* − *V* plane (b, d). For (a) and (b), *ϕ* = 0.15. Since *ℛ*_0_ = 0.84, the disease free equilibrium *E*_0_ is stable. For (c) and (d), *ϕ* has been decreased to 0.05 causing *ℛ*_0_ to increase to 1.18. Since *ℛ*_0_ > 1, the disease free equilibrium *E*_0_ is unstable and the endemic equilibrium *E*_1_ exists and is stable.

Furthermore, the endemic equilibrium *E*_1_ exists (and is locally asymptotically stable) if and only if *ℛ*_0_ > 1 (see Figure 2).

It is therefore important to analyze how *ℛ*_0_ changes in terms of model parameters in order to study methods to control the spread of the epidemic. For instance, when vaccination rates do not depend on benefits or costs (i.e., *k* or *α* is 0), there is a constant vaccination rate *ϕ*. Not surprisingly, as this rate increases, *ℛ*_0_ decreases and so the disease can be controlled by a sufficiently high vaccination rate. Constant vaccination rates correspond to involuntary vaccination programs, where the latter result is well-known in related models [28, 29].

**Figure 3:**
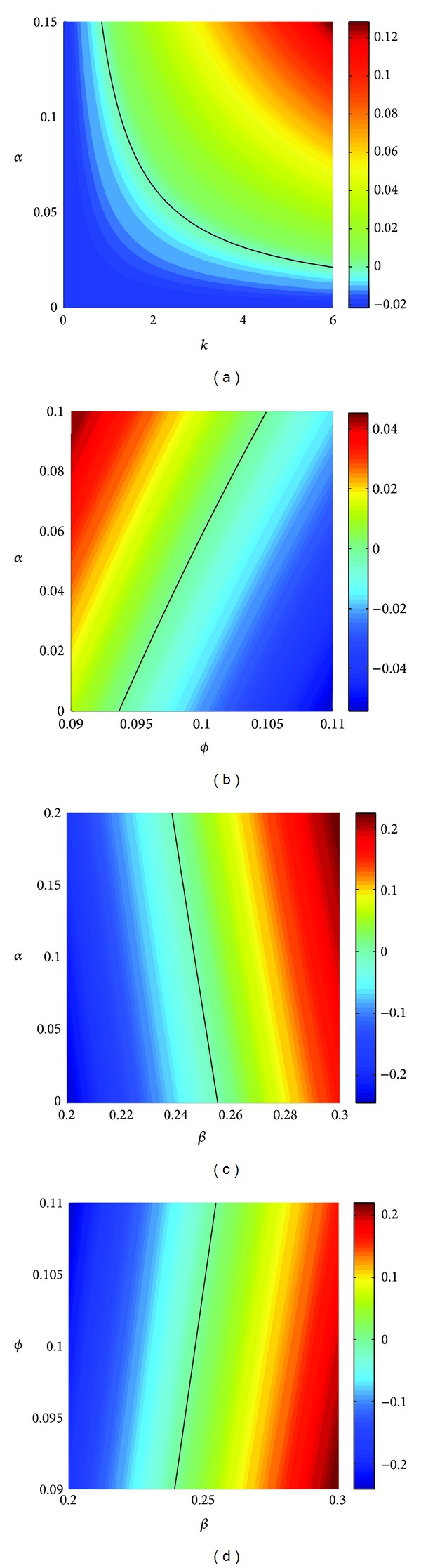
The logarithm log⁡*ℛ*_0_ of the basic reproduction number of system (1). Except as described below, the parameters used here are as follows: *A* = 0.5, *β* = 0.25, *q* = 0.9, *d* = 0.1, *r* = 0.1, *k* = 2, *α* = 0.1, and *ϕ* = 0.1. log⁡*ℛ*_0_ is shown (a) when the degree of rationality *k* is increased from 0 to 6 and the perceived cost of vaccination *α* is varied from 0 to 0.15; (b) when the decision rate *ϕ* is varied from 0.09 to 0.11 and *α* is varied from 0 to 0.1; (c) when the incidence rate *β* is varied from 0.2 to 0.3 and *α* is varied from 0 to 0.2; (d) when *β* is varied from 0.2 to 0.3 and *ϕ* is varied from 0.09 to 0.11. The solid curve in each panel denotes the points where the basic reproduction number *ℛ*_0_ is equal to 1 (i.e., log⁡*ℛ*_0_ = 0). The disease-free equilibrium is stable for parameter values corresponding to points under these curves and unstable for those above for (a), (b), and (c) and the opposite holds for (d). The column on the right-hand side of each panel gives the color coding for different values of log⁡*ℛ*_0_.

Of more importance for us, since we are interested in the effects of voluntary decisions concerning vaccinations, is how *ℛ*_0_ changes when *k* and *α* are both positive (as well as *ϕ* and *β*). For instance, for fixed *ϕ*, *β*, and *α*, *ℛ*_0_ increases as *k* increases (see Figure 3(a)). That is, as individuals become more precise in their estimates of benefits and costs (basing their decision whether or not to be vaccinated on which action has the higher payoff), their degree of rationality *k* may increase and cause the disease free equilibrium to become more unstable. There are a number of policy implications contained in this result. One implication is then that too much information in the general population may be bad for the control of an epidemic (a somewhat surprising outcome) unless other model parameters are also changed (e.g., the perceived cost of vaccination *α* is reduced). This outcome is examined more closely later in this section and policy initiatives to counteract it are discussed in the conclusions (Section 5).

The disease free equilibrium also becomes more unstable when *α* is increased (with other parameters fixed) (see Figures 3(a), 3(b), and 3(c)), but this is not so surprising since one would expect fewer susceptibles to be vaccinated if the cost of vaccination increases. On the other hand, as the percentage of susceptible individuals making the decision whether to be vaccinated per unit time increases (i.e., *ϕ* increases), the disease is better controlled (see Figure 3(b)). Put another way, this also says that for diseases that progress at a slower time-scale (e.g., through a lower incidence rate *β*), lower decision rates *ϕ* on vaccination can still be effective in controlling the disease (everything else being equal) (see Figure 3(d)). This is also a well-known result [28, 29] for related models with constant (involuntary) vaccination rates *φ*(*S*, *I*, *R*, *V*) = *ϕ*.

**Figure 4:**
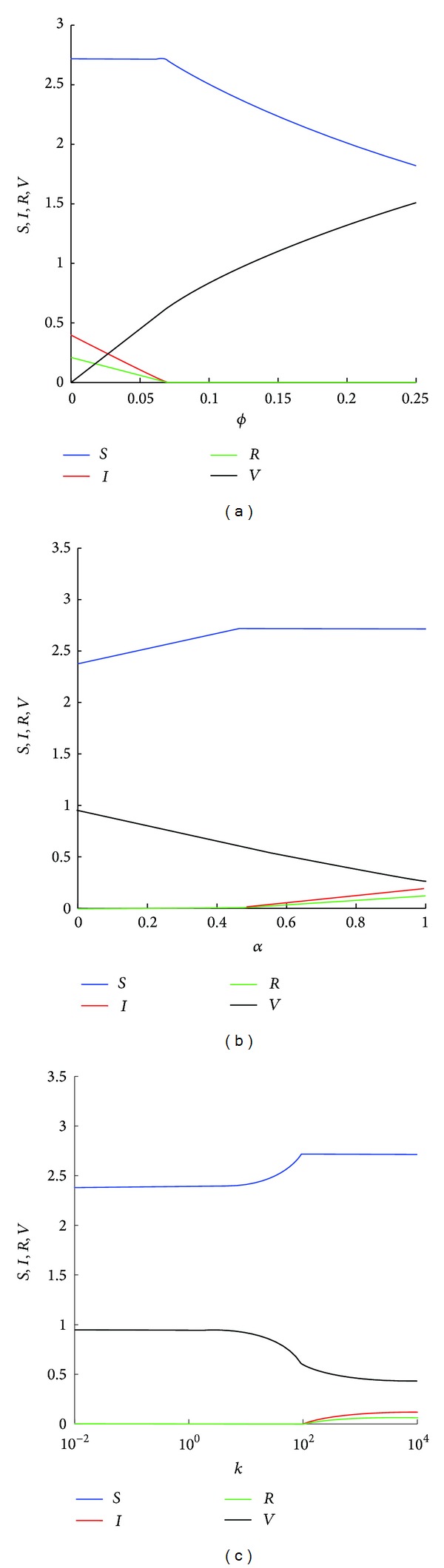
Bifurcation diagrams showing the equilibrium structure of system (1). Except as described below, the parameters used here are as follows: *A* = 0.5, *β* = 0.25, *q* = 0.9, *d* = 0.15, *r* = 0.08, *k* = 2, *α* = 0.01, and *ϕ* = 0.12. With these parameters, *ℛ*_0_ = 0.8786. (a) *ϕ*, the proportion of susceptible individuals who are faced with the decision of whether or not to be vaccinated per unit time, is varied from 0 to 0.25; (b) *α*, the perceived cost of taking the vaccination, is varied from 0 to 1; (c) *k*, the degree of rationality, is varied from 0.01 to 10^4^.

Similar results can also be obtained from the bifurcation diagram (see Figure 4); that is, increasing the rate *ϕ* at which decisions are made or decreasing the cost *α* of vaccination are both effective means in slowing down the spread of an epidemic. However, with the increase in the amount of information individual decision-makers have (reflected by an increase in *k*), the chances that an epidemic spreads actually increase.

In order to further discuss the effect of *k* on the spread of an epidemic, we compare the general smoothed best response for *k*, a fixed positive parameter, to an extreme situation, the best response (i.e., *k* → *∞*). For the best response, when *W*_*p*_ > *W*_*n*_ (i.e., *βI*/(*S*+*I*+*R*+*V*)^*q*^ > *α*), we have *b*(*W*) = 1, indicating that the per individual rate of vaccination uptake is *ϕ*. Thus system (1) can be written as

(18) S˙=A−βSI(S+I+R+V)q−dS−Sϕ,I˙=βSI(S+I+R+V)q−rI−dI,R˙=rI−dR,V˙=Sϕ−dV,α<βI(S+I+R+V)q,

with basic reproduction number *ℛ*_*Bp*0_ = *Aβ*/((*r* + *d*)(*d* + *ϕ*)(*A*/*d*)^*q*^). When *W*_*p*_ < *W*_*n*_, we have *b*(*W*) = 0, and thus the per individual rate of vaccination uptake is 0. In this case, system (1) becomes

(19) S˙=A−βSI(S+I+R+V)q−dS,I˙=βSI(S+I+R+V)q−rI−dI,R˙=rI−dR,V˙=−dV,α>βI(S+I+R+V)q.

The basic reproduction number of system (19) is *ℛ*_*Bn*0_ = *βA*/((*A*/*d*)^*q*^(*r* + *d*)*d*). Here we consider the case when the disease becomes endemic without vaccination (i.e., *ℛ*_*Bn*0_ > 1) but can be controlled with a sufficiently high constant vaccination rate *ϕ* (i.e., with a properly chosen *ϕ*, the basic reproduction number of model (18) *ℛ*_*Bp*0_ is less than 1). Hence, the disease-free equilibrium of subsystem (18), *E*_*Bp*0_ = (*A*/(*d* + *ϕ*), 0, 0, *ϕA*/(*d*(*d* + *ϕ*))), is globally asymptotically stable, and the endemic equilibrium of the subsystem (19), *E*_*Bn*1_ = (*S*_*Bn*1_*, *I*_*Bn*1_*, *R*_*Bn*1_*, *V*_*Bn*1_*), where

(20) SBn1∗=(r+d)(A/d)qβ,IBn1∗=Ar+d−(A/d)qdβ,RBn1∗=rAd(r+d)−r(A/d)qβ,VBn1∗=0,

is globally asymptotically stable (see the Appendix).

The discussion on the behavior of models (18) and (19) is divided into the following three cases depending on the cost *α* of being vaccinated. When there is no cost of vaccination (*α* = 0, Case 1), we have *W*_*p*_ > *W*_*n*_ as long as the number of infected is not 0. Thus the epidemic is described by system (18) which evolves to the disease-free equilibrium *E*_*Bp*0_.

On the other hand, for any positive cost of vaccination, the disease-free equilibrium is unstable and a stable endemic equilibrium that depends on the cost level exists as shown in the appendix. For high costs (specifically, for *α* > *Aβ*/((*r* + *d*)(*A*/*d*)^*q*^) − *d*, Case 2), it is shown there that *E*_*Bn*1_ is stable. We note that *Aβ*/((*r* + *d*)(*A*/*d*)^*q*^) − *d* > 0 is guaranteed by *ℛ*_*Bn*0_ > 1.

For intermediate costs (specifically, for 0 < *α* < *Aβ*/((*r* + *d*)(*A*/*d*)^*q*^) − *d*, Case 3), the endemic equilibrium has a lower proportion of infected:

(21) $$\begin{array}{c} I_{B1}^{*}=\frac{\alpha {\left(A/d\right)}^{q}}{\beta } \end{array}$$

than that given by *I*_*Bn*1_* in (20). In fact, *I*_*B*1_* can also be obtained from (11) by taking the limit

**Figure 5:**
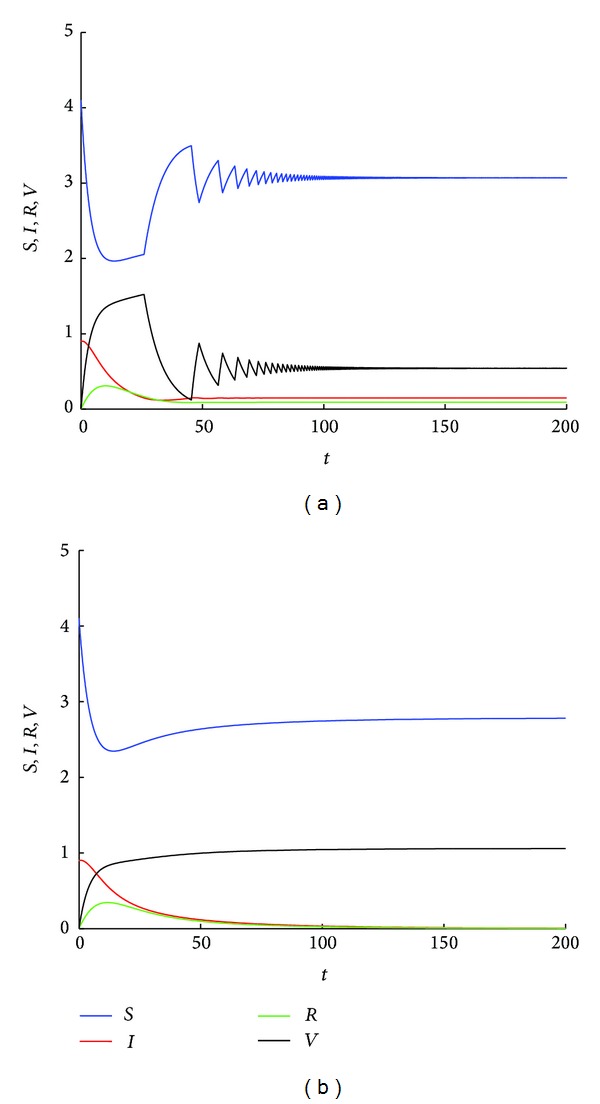
(a) Simulated results of the best-response systems (18) and (19) for *A* = 0.5, *β* = 0.23, *q* = 0.9, *d* = 0.13, *r* = 0.08, *ϕ* = 0.1, and *α* = 0.01. With these parameters, the basic reproduction number of models (18) and (19) are *ℛ*_*Bp*0_ = 0.7083 and *ℛ*_*Bn*0_ = 1.2532, respectively. Since *α* < *Aβ*/((*r* + *d*)(*A*/*d*)^*q*^) − *d* = 0.0329, the epidemic dynamics switch between models (18) and (19) until an endemic equilibrium is reached. (b) Simulated results of system (1) for *k* = 2 with all the other parametric values are the same as (a). Since the basic reproduction number *ℛ*_0_ = 0.9076, the disease free equilibrium *E*_0_ of model (1) is stable.

(22) IB1∗=lim⁡k→∞I1∗=lim⁡k→∞(A/d)q(kα+2Q)kβ=α(A/d)qβ+2  (A/d)qβlim⁡k→∞Qk

and showing that lim⁡_*k*→*∞*_(*Q*/*k*) = 0 (see the Appendix). It is also interesting to note that, in this last case, the epidemic dynamics will continue to switch between the two systems (18) and (19), driven by the best response based vaccine uptake (see Figure 5(a)).

The above discussion indicates that, when susceptibles make vaccination decisions based on the best response, the disease-free equilibrium is unstable when *α* > 0. In fact, the best response correspondence can then be approximately obtained by letting *k* → *∞* in the smoothed best response. Thus, the basic reproduction number of systems (18) and (19) can be calculated by taking the limit

(23) $$\begin{array}{c} {ℛ}_{B0}=\underset{k\to \infty }{\lim}{ℛ}_{0}=\frac{\beta A}{{\left(A/d\right)}^{q}\left(r+d\right)\;d}. \end{array}$$

We notice that *ℛ*_*B*0_ = *ℛ*_*Bn*0_, indicating that an epidemic cannot be totally eliminated if each individual adopts the best response (see Figure 5(a)).

However, under the smoothed best response, the per individual rate of vaccination uptake is still positive even when 0 < *βI*/(*S*+*I*+*R*+*V*)^*q*^ < *α*, and so the number of infected can continue to decrease. In fact, the disease-free equilibrium may be locally asymptotically stable. For properly chosen *ϕ* and *k*, the epidemic can be eliminated (see Figure 5(b)). That is, the smoothed best response is more effective in controlling the disease than the best response. Generally, the basic reproduction number *ℛ*_0_ is an increasing function of *k*, indicating that the more sensitive the susceptible population is to the payoff difference, the more difficult it is to control the initial spread of the disease.

In Case 1 (i.e., *α* = 0), we have shown that the disease dies out under the best response when *ℛ*_*Bp*0_ < 1. For the smooth best response with *k* bounded, the epidemic model is given by (1). The corresponding basic reproduction number can be obtained by substituting *α* = 0 into (8), which yields

(24) $$\begin{array}{c} {ℛ}_{\alpha 0}=\frac{\;\beta A}{{\left(A/d\right)}^{q}\left(r+d\right)\left(\;d+\left(1/2\right)\phi \right)}. \end{array}$$

**Figure 6:**
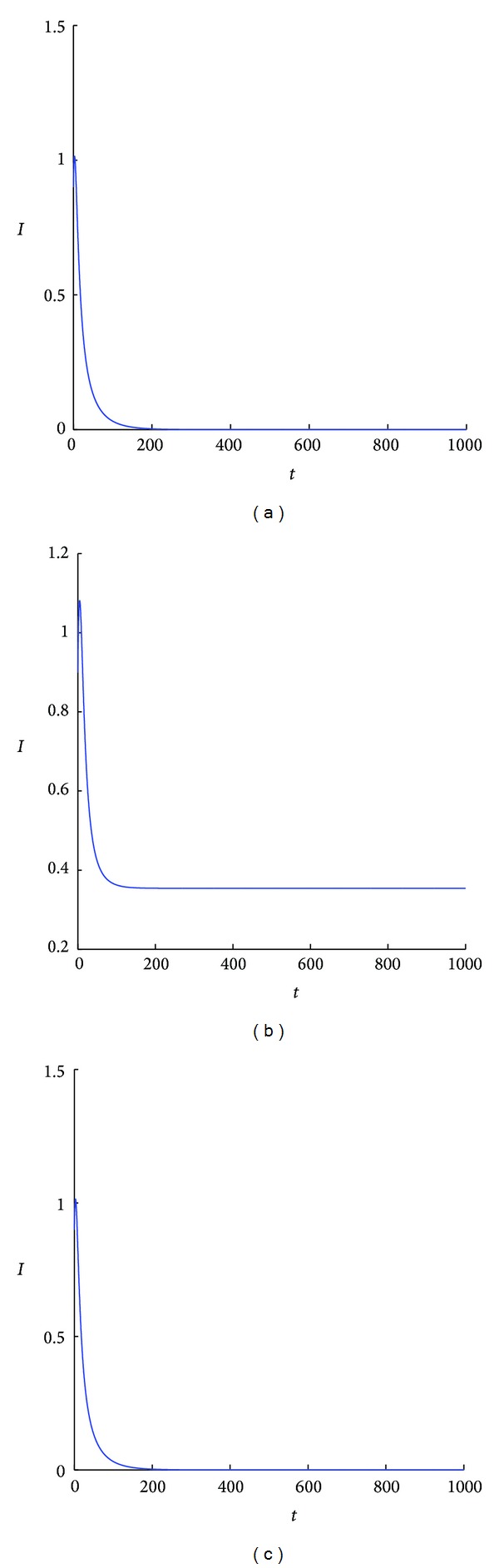
(a) Simulated results of the best-response systems (18) and (19) for *A* = 0.5, *β* = 0.3, *q* = 0.9, *d* = 0.1, *α* = 0, *ϕ* = 0.1, and *r* = 0.1. The system is converging to the disease-free equilibrium. (b) Simulated results of system (1) for *k* = 2 with all the other parametric values the same as (a). (c) Simulated results of system (1) for *k* = 200000 with all the other parametric values as (a). All these systems have the same disease-free equilibrium. In (c), the system is converging to an endemic equilibrium that is very close to this disease-free equilibrium and so the number of infected is almost 0.

We notice that the condition *ℛ*_*Bp*0_ < 1 does not guarantee that *ℛ*_*α*0_ < 1. Thus, the smoothed best response is not as effective as the best response with respect to epidemic control when *α* = 0 (see Figure 6). That is to say, if there is no “cost” to take the vaccine, the disease might be endemic if vaccination decisions are based on the smoothed best response in cases when the epidemic can be controlled under the best response. Furthermore, as seen in Figure 6(c), the number of infected at the endemic equilibrium decreases to 0 as *k* → *∞*, illustrating again that the epidemic model with best response is the limiting case as *k* → *∞* of the outcome for the smoothed best response.

## 5. Conclusions

In this paper, the smoothed best response correspondence is used to model a game-theory based vaccination uptake decision during an epidemic. It is assumed that each individual is rational and follows a cost-benefit analysis to make decisions on vaccination uptake. We obtain the basic reproduction number of the model and investigate how the sensitivity of these decisions to differences in costs and benefits affects the spread and control of the epidemic. The effect of vaccination decisions based on the best response (that assumes complete and accurate information on costs and benefits) is also analyzed and compared to that based on the smoothed best response.

Our investigation indicates that, when the “cost” of taking the vaccination is positive, the smoothed best response is more effective controlling the disease than the best response. As mentioned in Section 4, this result suggests a number of policy implications. As the amount of information available to the population on the risks of being infected and the risks *α* associated with the vaccine increase, it is important that *α* be made as small as possible compared to the infection risk. Besides making vaccines safer, policy makers can emphasize the benefits of vaccination to those susceptibles who have higher risk of infection in order to convince them to be vaccinated. Although this is beyond the scope of our investigation since we assume each epidemiological class is homogeneous (in particular, all susceptibles have the same risk of infection), this is an important direction of future research.

Secondly, the social benefits of being vaccinated can be emphasized so that individuals obtain positive payoff effects associated with the public health benefits of vaccinations. Such initiatives have the potential to counteract the free riding problem and drive perceived vaccination costs to zero (or perhaps even negative). As we have shown, when there is no cost to take the vaccine, the best response becomes superior to the smoothed best response in controlling the disease.

In general, rational decision-making by individuals is based on up-to-date information about the spread of an epidemic as well as about the “cost” of vaccination. These two types of information have opposite effects since knowledge about the spread of an epidemic encourages individuals to take the vaccine, while the information about the “cost” discourages them (if the cost is positive). Hence, with respect to the control of an epidemic, (smoothed) best response-based vaccination decisions might not be as effective as compulsory vaccination programs with constant vaccination rates. However, compulsory programs may not be possible in this information age where individuals want to avoid taking unnecessary vaccine with potential side effects. In such scenarios, it becomes more important to understand how individual rational decisions based on game theory affect the spread of a disease.

## Appendix

We first calculate the basic reproduction number for system (1). Using the standard notation of the next generation method [27], we have

(A.1) $$\begin{array}{c} ℱ=\frac{\beta SI}{{\left(S+I+R+V\right)}^{q}},\;\;𝒱=rI+dI. \end{array}$$

It follows from (A.1) that

(A.2) $$\begin{array}{c} 𝔽=\frac{2\beta A}{{\left(A/d\right)}^{q}\left(2d+\phi \left(1-\tanh\left(\left(1/2\right)k\alpha \right)\right)\right)},\\ 𝕍=r+d. \end{array}$$

The basic reproduction number is then obtained as (in general, *ρ*(*𝔽𝕍* ^−1^) is the spectral radius of the matrix *𝔽𝕍* ^−1^; since *𝔽𝕍* ^−1^ is a 1 × 1 matrix with a positive entry, *ρ*(*𝔽𝕍* ^−1^) equals this entry)

(A.3) ℛ0=ρ(𝔽𝕍−1)=2βA(A/d)q(r+d)(2d+ϕ(1−tanh((1/2)kα)))

which is (8) in the main text.

We now discuss the behavior of the best response (i.e., models (18) and (19)) when the cost of vaccination is positive (i.e., *α* > 0). First assume that we are in Case 2 (i.e., *α* > *Aβ*/((*r* + *d*)(*A*/*d*)^*q*^) − *d*). When the initial value of this system satisfies

(A.4) $$\begin{array}{c} \frac{\beta I_{0}}{{\left(S_{0}+I_{0}+R_{0}+V_{0}\right)}^{q}}<\alpha , \end{array}$$

we have *W*_*p*_ < *W*_*n*_, indicating that the epidemic dynamics is described by model (19). Since the endemic equilibrium of subsystem (19), *E*_*Bn*1_, is globally asymptotically stable, the trajectory is convergent to this equilibrium point. At *E*_*Bn*1_, the benefit of taking the vaccine becomes *βI*_*Bn*1_/(*S*_*Bn*1_+*I*_*Bn*1_+*R*_*Bn*1_+*V*_*Bn*1_)^*q*^ = *Aβ*/((*r* + *d*)(*A*/*d*)^*q*^) − *d* < *α*. Since *W*_*p*_ < *W*_*n*_, the vaccine uptake rate is 0 (i.e., the epidemic dynamics is still governed by system (19)). Hence the trajectory of the model converges to the equilibrium *E*_*Bn*1_.

On the other hand, if the initial condition of the systems (18) and (19) satisfies

(A.5) $$\begin{array}{c} \frac{\beta I_{0}}{{\left(S_{0}+I_{0}+R_{0}+V_{0}\right)}^{q}}>\alpha , \end{array}$$

we have *W*_*p*_ > *W*_*n*_ at the start. According to the best response, the epidemic dynamics is initially described by system (18). Since *ℛ*_*Bp*0_ < 1, the number of infected will decrease. If the vaccination uptake remained at *ϕ*, the trajectory would be attracted to the disease-free equilibrium *E*_*Bp*0_. However, under the best response, with the decrease of *I*, once *βI*/(*S* + *I* + *R* + *V*)^*q*^ < *α* (i.e., *W*_*p*_ < *W*_*n*_), the vaccine uptake rate becomes 0. Then the epidemic dynamics is described by subsystem (19). Since *E*_*Bn*1_ is globally asymptotically stable and *α* > *Aβ*/((*r* + *d*)(*A*/*d*)^*q*^) − *d*, the trajectory is convergent to the equilibrium *E*_*Bn*1_. Thus, in Case 2, *E*_*Bn*1_ is globally asymptotically stable.

Now assume that we are in Case 3 (i.e., 0 < *α* < *Aβ*/((*r* + *d*)(*A*/*d*)^*q*^) − *d*). Driven by the best response, the epidemic dynamics now switches between models (18) and (19). When *βI*/(*S*+*I*+*R*+*V*)^*q*^ > *α*, since the benefit of taking a vaccine is higher than the cost, the epidemic spreads according to model (18). Because *ℛ*_*Bp*0_ < 1, the number of infected decreases. With the decrease of *I*, an individual's chance of being infected also decreases. As soon as *βI*/(*S*+*I*+*R*+*V*)^*q*^ < *α*, according to the best response, a rational individual should not take the vaccine. Thus, the epidemic dynamics is described by (19). Since *ℛ*_*Bn*0_ > 1 and the number of infected is not 0 (since the switch starts as soon as *I* < *α*(*S*+*I*+*R*+*V*)^*q*^/*β*), the number of infected increases. With the increase of *I*, as soon as *βI*/(*S*+*I*+*R*+*V*)^*q*^ > *α*, under the best response, the epidemic spread will be governed by system (18) again. The epidemic dynamics will continue to switch between the two systems (18) and (19), driven by the best response based vaccine uptake. From the numerical simulations (e.g., Figure 5(a)) eventually an equilibrium will be reached with *I* = *α*(*S*+*I*+*R*+*V*)^*q*^/*β*. Since  *S* + *I* + *R* + *V* = *A*/*d* as *t* → *∞*, the endemic equilibrium satisfies

(A.6) $$\begin{array}{c} I_{B1}^{*}=\frac{\alpha \;{\left(A/d\right)}^{q}}{\beta }. \end{array}$$

The main text claims that (A.6) can also be obtained from (11) by taking the limit

(A.7) IB1∗=lim⁡k→∞I1∗=lim⁡k→∞(A/d)q(kα+2Q)kβ=α(A/d)qβ+2(A/d)qβlim⁡k→∞Qk.

To see this, consider lim⁡_*k*→*∞*_(*Q*/*k*). Recall that *Q* is the intersection of the following two functions:

(A.8) $$\begin{array}{c} f_{1}\left(x\right)=\frac{1}{{\text{e}}^{2\;x}+1},\\ f_{2}\left(x\right)=\frac{P_{2}}{{\left(A/d\right)}^{q}k\phi \left(r+d\right)}+\;\frac{2}{\phi k}x. \end{array}$$

As *k* → *∞*, the second equation of (A.8) is reduced to

(A.9) $$\begin{array}{c} f_{2}\left(x\right)=\frac{P_{2}}{{\left(A/d\right)}^{q}k\phi \left(r+d\right)}, \end{array}$$

indicating that *Q* is the solution of

(A.10) $$\begin{array}{c} \frac{1}{{\text{e}}^{2x}+1}=\frac{P_{2}}{{\left(A/d\right)}^{q}k\phi \left(r+d\right)}:=M. \end{array}$$

There is a unique solution to (A.10) if and only if 1 > *M* > 0. We note that *M* = (*d* + *α*)/*ϕ* + 1 − *Aβ*/((*A*/*d*)^*q*^ *ϕ*(*r* + *d*)), which does not depend on *k*. It is not difficult to show that *M* > 0 if and only if *Aβ*/((*A*/*d*)^*q*^(*d* + *α* + *ϕ*)(*r* + *d*)) < 1, which is guaranteed by the assumption that *ℛ*_*Bp*0_ < 1. Next, we show that *M* < 1. It is easy to verify that *M* < 1 is equivalent to (*d* + *α*)/*ϕ* − *Aβ*/((*A*/*d*)^*q*^ *ϕ*(*r* + *d*)) < 0, which is guaranteed by the condition of Case 3; that is,

(A.11) $$\begin{array}{c} 0<\alpha <\frac{A\beta }{\left(r+d\right){\left(A/d\right)}^{q}}-d. \end{array}$$

The solution to (A.10) can then be obtained as *Q* = (1/2)ln⁡((1/*M*) − 1), implying that

(A.12) $$\begin{array}{c} \underset{k\to \infty }{\lim}\frac{Q}{k}=0. \end{array}$$

Thus, it follows from (A.7) that

(A.13) $$\begin{array}{c} I_{B1}^{*}=\underset{k\to \infty }{\lim}I_{1}^{*}=\frac{\alpha {\left(A/d\right)}^{q}}{\beta }. \end{array}$$

Next, we prove the global stability of the endemic equilibrium of model (19). It follows from $\dot{V}=-dV$ that lim⁡_*t*→*∞*_ *V* = 0. Thus, when there is no game theory involved, system (19) is reduced to

(A.14) $$\begin{array}{c} \dot{S}=A-\frac{\beta SI}{{\left(S+I+R\right)}^{q}}-dS,\\ \dot{I}=\frac{\beta SI}{{\left(S+I+R\right)}^{q}}-rI-dI,\\ \dot{R}=rI-dR. \end{array}$$

We note that the equation for *R* in the above system is decoupled from other equations and as such we only need to consider the dynamical behavior of the first two equations of system (A.14). We construct the following Lyapunov function:

(A.15) U(S,I)=(SSBn1∗−1−ln⁡(SSBn1∗)) +IBn1∗SBn1∗(IIBn1∗−1−ln⁡(IIBn1∗))

that has a unique minimum at (*S*_*Bn*1_*, *I*_*Bn*1_*). The derivative of *U* along the solutions of model (A.14) is

(A.16) U˙=S−S∗SSBn1∗S˙+IBn1∗SBn1∗I−I∗II∗I˙=S−SBn1∗SSBn1∗(A−βSI(S+I+R)q−dS) +IBn1∗SBn1∗I−IBn1∗IIBn1∗(βSI(S+I+R)q−rI−dI).

Using lim⁡_*t*→*∞*_(*S* + *I* + *R*) = *A*/*d*, we have

(A.17) U˙=S−SBn1∗SSBn1∗(−β  SI−SBn1∗  IBn1∗(A/d)q−d(S−SBn1∗)) +IBn1∗SBn1∗I−IBn1∗IIBn1∗(βSI−SBn1∗  IBn1∗(A/d)q−(r+d)(I−IBn1∗))=−(IBn1∗SSBn1∗(A/d)q+dSSBn1∗)(S−SBn1∗)2.

Thus, every trajectory of (A.14) converges to an invariant subset of {(*S*, *I*, *R*) | *S* = *S*_*Bn*1_*} by LaSalle's invariance principle [30]. Since $\dot{S}=0$ in this set, it is clear that the only such invariant subset is the endemic equilibrium *E*_*Bn*1_ and so *E*_*Bn*1_ is globally asymptotically stable.
